# Dysregulation of PI3K/Akt/PTEN Pathway in Canine Mammary Tumor

**DOI:** 10.3390/ani11072079

**Published:** 2021-07-12

**Authors:** Soo-Hyeon Kim, Byung-Joon Seung, Seung-Hee Cho, Ha-Young Lim, Min-Kyung Bae, Jung-Hyang Sur

**Affiliations:** Department of Veterinary Pathology, College of Veterinary Medicine, Konkuk University, Seoul 05025, Korea; windsl@naver.com (S.-H.K.); bjseung@naver.com (B.-J.S.); shchopa11@naver.com (S.-H.C.); hylim07@gmail.com (H.-Y.L.); mkbae4136@naver.com (M.-K.B.)

**Keywords:** dogs, mammary neoplasms, PI3K/Akt/PTEN pathway, PIK3CA H1047R mutation

## Abstract

**Simple Summary:**

The importance of the PI3K/Akt/PTEN pathway in canine mammary tumor has recently been highlighted due to the finding of frequent PIK3CA mutation. Effect of PIK3CA mutation to expression of downstream molecules has never been investigated in canine mammary tumors. We found that frequency of PIK3CA H1047R mutation was 14.3% using Sanger sequencing in canine mammary tumors, and downstream molecules Akt2, p-Akt, and PTEN were dysregulated in mammary tumors when compared to normal mammary gland. However, we could not find any significant relevance between PIK3CA H1047R mutation and expression of downstream molecules, except a paradoxical higher level of PTEN in PIK3CA-mutated tumors. We suggest that dysregulation of PI3K/Akt/PTEN pathway components is a feature in canine mammary tumors, but not directly associated with PIK3CA H1047R mutation except for the expression of PTEN. Further study is necessary to find the role of PIK3CA mutation in the regulation of downstream molecules to improve our understanding and therapeutic approach for canine mammary tumors.

**Abstract:**

The PI3K/Akt/PTEN axis is one of the most important signaling pathways in tumorigenesis. Recently, mutation of PIK3CA has been highlighted due to the similarities of mutational hotspots in both dogs and humans. PIK3CA H1047R (c.3140A > G) has been discovered as the most common mutational hot spot in canine mammary tumor in recent studies, while the feature of PIK3CA-mutated canine mammary tumor is obscure. Methods: A total of 83 mammary samples classified as normal (*n* = 13), adenoma (*n* = 25), low-grade carcinoma (*n* = 21), and high-grade carcinoma (*n* = 24) were included in this study. Genomic DNA from each sample was extracted, amplified by conventional PCR, and analyzed through Sanger sequencing. Analysis for the expression of PIK3CA, Akt, p-Akt, and PTEN was performed by immunohistochemistry, and of Akt2 by RNA in situ hybridization. Results: PIK3CA H1047R mutation was detected in 14.3% (10/70) of tumor samples. Dysregulation of p-Akt, Akt2, and PTEN was observed in mammary tumor samples, but only PTEN dysregulation was associated with PIK3CA H1047R mutation. Conclusions: The present study showed that dysregulation of components in the PI3K/Akt/PTEN pathway is a feature of canine mammary tumors, but this dysregulation is not directly correlated to the PIK3CA H1047R mutation except for PTEN expression.

## 1. Introduction

PI3K (phosphoinositide-3-kinase) was first discovered as a viral oncoprotein that phosphorylates phosphatidylinositol, induces the transformation of cells, and has been revealed to be conserved in mammals [1].

PIK3CA is one of the catalytic subunits of the class I PI3K subfamily and encodes a catalytic subunit p110α that functions as a heterodimer with the p85 regulatory subunit [2]. Class I PI3K is stimulated by numerous signals received from tyrosine kinase receptors, cytokines, and G protein-coupled receptors [3,4]. In response to the signals, PI3K phosphorylates lipids in plasma membrane, phosphatidylinositol-4,5-biphosphate to phosphatidylinositol-3,4,5-trisphosphate [3,4]. The lipids that are produced in this reaction interacts with the v-Akt murine thymoma viral oncogene homolog (Akt) pleckstrin homology domain, and consequently, phosphorylated Akt plays a key second messenger to various cell signaling [3,4]. In addition, the phosphatase and tensin homolog deleted on chromosome 10 (PTEN) dephosphorylates the lipid phosphatidylinositol-3,4,5-trisphosphate, which is the product of PI3K, thus hindering the activation of Akt and acting as a tumor suppressor [5]. 

Members of the PI3K family play active roles in a wide range of physiologic processes, thus making the dysregulation reasonable in several diseases including diabetes, neurological, and immunological disorders [6]. The role of PI3K has been highlighted especially in oncogenesis, because overactivation of PI3K with enhanced Akt activity and PTEN suppression is associated with most hallmarks of cancer [6,7]. For instance, overactivation of the PI3K/Akt pathway can induce the progression of cell cycle and cellular proliferation through stability regulation of cyclin D and p21^Cip1^ and can inhibit apoptosis by modulating the activity of Bcl-2 family members [8]. Moreover, PI3K signaling contributes to cell migration and migratory cell polarization in various cell types [9]. Overall, the frequent activation of the PI3K pathway with diverse contribution to oncogenesis makes it an attractive therapeutic target [10].

Specific mutations in PI3KCA have been identified in various tumors from 2004 [1,11,12], and intense studies investigating the role and regulation of PIK3CA have been progressed. Mutations in the PIK3CA gene were found in a wide range of human cancers including glioblastoma [12,13], gastric cancer [12,14], lung cancer [12,15], colorectal cancer [16], and breast cancer [11,12]. In human breast cancer, somatic mutation of PIK3CA has been found in 8–40% of case samples. Mutational hotspots were identified on exon 9 and exon 20, and the most frequent mutation has been exon 20 H1047R in human breast cancer, implicating that it is an oncogenic driver [11,12,17,18,19,20].

Several investigations have proven how mutant PIK3CA H1047R specifically influence oncogenic and physiological processes in in vitro and in vivo models. The PIK3CA H1047R mutation has been known to gain-of-function mutation stimulating catalytic activity [21], and expression of PIK3CA H1047R induced tumor initiation [22], cell dedifferentiation [23], tumor heterogeneity [24], and invasiveness and migration in mammary tumor cells [25].

In recent years, research applying next-generation sequencing has opened a new landscape in veterinary oncology. PIK3CA has started to be highlighted because its frequent mutations in canine tumors have been revealed. The latest studies have shown that single missense mutation H1047R is the feature that is most discovered in canine hemangiosarcoma [26] and mammary tumors [26,27,28].

There are few studies exploring the expression patterns of the PI3K pathway in canine mammary tumors, reporting the upregulation of phospho-Akt (p-Akt) and loss of PTEN is related to histologic and clinical malignancy [29,30]. However, research examining the correlation between H1047R mutation and the expression of PI3K/Akt pathway molecules is missing in the literature. Therefore, the current study aims to analyze the frequency of the PIK3CA H1047R mutation in canine mammary tumors and to investigate the correlation between PIK3CA H1047R mutation and the factors including the PI3K/Akt/PTEN pathway components’ expression, histopathological features, and clinical characteristics.

## 2. Materials and Methods

### 2.1. Ethical Statement

All samples were surgically resected for treatment from the diseased dogs and submitted for histopathological diagnosis, and live laboratory animals were not used in this study. Therefore, Institutional Animal Care and Use Committee (IACUC) approval was waived. Informed consent for using the surgically excised tissues were obtained from the owners of dogs.

### 2.2. Sample Collection and Histopathological Evaluation

Canine mammary gland tissues were surgically removed for treatment and submitted for histopathological diagnosis from local animal hospitals. Mammary gland samples were archived during 2020 and those archived tissues were retrieved. Tissues were fixed in 10% formalin and embedded in paraffin wax. Tissue blocks were made into 4 μm slides and stained with hematoxylin and eosin for histopathological examination. Histopathological evaluation including histologic grading and subtypes of tumors was conducted based on the classification system of Goldschmidt et al. [31]. Tissue samples were divided into four classes: normal or hyperplastic mammary gland, mammary adenoma, low-grade mammary carcinoma, and high-grade mammary carcinoma. In addition, the presence of lymphatic invasion and the number of mitotic figures in 10 high-power fields (2.37 mm^2^) were also evaluated. Clinical data including age, spay, and size of tumors were also collected in cases available.

### 2.3. DNA Extraction

FFPE tissue samples were serially sectioned, and the first section of serial sections was hematoxylin and eosin stained. Stained slides were evaluated under a microscope, and the parts comprised only of neoplastic mammary cells were marked. Marked parts of sections were carefully picked up by trimming the rest of marked parts with a surgical blade. Genomic DNA was extracted using a QIAamp DNA FFPE Tissue Kit (Qiagen, Hilden, Germany) from those marked parts of each section.

### 2.4. PCR and Sequence Analysis

PCR was performed with a commercial PCR Premix Kit (Maxime PCR PreMix Kit, iNtRON Biotechnology, Seoul, Korea). The primer set (Forward: 5′-CCCCAGAAGGCCTCTCTAAT-3′; Reverse: 5′-TGCAATCAGTCTTTGCCTGT-3′) was used to detect the c.3140A > G (H1047R) missense mutation in the coding sequence of canine PIK3CA (NCBI reference: NC_006616.4). The final volume of the PCR mix contained 2.5 u Taq DNA polymerase, 2.5 mM of each dNTPs, 2 μL of genomic DNA, 10 pmol of forward and reverse primers, and distilled water. PCR cycling condition was as followed: initial denaturation at 95 °C for 3 min, 35 cycles of 95 °C for 30 s, 58 °C for 30 s, 72 °C for 1 min, and final extensions at 72 °C for 5 min. PCR products were electrophoresed, and specific bands were excised from the agarose gel. Amplified PCR products were extracted from the gel using a QIAquick Gel Extraction Kit (Qiagen, Hilden, Germany). Extracted DNA was submitted to Bionics Co. (Seoul, Korea) for Sanger sequencing. Sequencing was conducted for both the forward and reverse primer. The results were analyzed utilizing open-source software BioEdit 7.2.

### 2.5. Immunohistochemistry (IHC)

#### 2.5.1. Controls for IHC

External and internal positive controls and isotype negative controls were performed to validate the primary antibodies for canine use [32]. For external positive controls, canine normal kidney tissue was applied with PIK3CA, PTEN, and Akt antibodies [33,34], and canine hemangiosarcoma was used for the positive control of the p-Akt antibody [35]. Internal negative controls were performed by applying the PIK3CA and p-Akt antibody substituted with rabbit polyclonal antibody, replacing the Akt antibody with the rabbit IgG antibody and PTEN antibody with the mouse IgG1 kappa antibody.

#### 2.5.2. IHC

FFPE tissues were sliced into 4 μm and picked up with silane-coated slides. Sections were dewaxed in 2-series of xylene, rehydrated in graded ethanol, and phosphate-buffered saline (PBS). Endogenous hydrogen peroxidase activity was quenched in 3% hydrogen peroxide for 20 min. Next, sections were boiled in a pressure cooker in citric acid buffer (pH 6.0) or Tris-EDTA buffer (pH 9.0) for different durations (Table S1). Slides were cooled down in iced water and incubated with 5% normal goat serum for 30 min at room temperature to block non-specific binding of antibodies. Slides were then incubated with primary antibodies (Table S1) and washed in PBS for 2 min, 3 times. After washing, sections were incubated with peroxidase-labelled anti-rabbit/mouse secondary antibody (Agilent, Santa Clara, CA, USA). Sections were again washed for 2 min in PBS three times and signals were visualized by 3,3′-diaminobenzidine. Tissue slides were washed in deionized water, counterstained in Gill’s hematoxylin, and dehydrated in graded alcohols.

### 2.6. RNA In Situ Hybridization (RISH)

Expression of Akt serine/threonine kinase 2 (Akt2) was analyzed using RISH instead of immunohistochemistry to avoid non-specific results due to the high similarities of amino acid sequences between three Akt isotypes [36]. RISH was conducted using the commercial kit (RNAscope, Advanced Cell Diagnostics, Newark, CA, USA) Concisely, FFPE tissues were sectioned into 4-μm on SuperFrost Plus adhesion slides (Thermo Fisher Scientific, Waltham, MA, USA) and were baked in 60 °C for 1 h. Tissue slides were then deparaffinized in xylene twice for 5 min each, washed in 100% ethanol twice for 1 min each, and air-dried. After deparaffinization, endogenous hydrogen peroxidase activity was quenched and slides were boiled in target retrieval solution for 15 min on a hot plate, with the temperature adjusted from 93 to 98 °C. For probe penetration, protease was applied onto the tissues and incubated in 40 °C for 30 min. Thereafter, sections were washed in distilled water, Akt2 probe (NCBI reference: NM_001012340.1) was hybridized, and the signal was amplified and developed by reacting with 3′3-diaminobenzidine. Slides were counterstained in 50% Gill’s hematoxylin. To examine whether RNA level is intact in FFPE tissues, the endogenous housekeeping gene UBC (NCBI reference: XM_038436353.1) was hybridized to serial sections of each sample.

### 2.7. Evaluation of IHC and RISH

Evaluation criteria for each immunohistochemical markers and RISH was described in Table S2. Evaluation criteria were referred from previous studies investigating each molecule [37,38,39,40].

### 2.8. Statistical Analysis

Evaluation data for immunohistochemistry and RISH were statistically analyzed through SPSS 25 (IBM corporation, New York, NY, USA) and graphs were drawn using GraphPad Prism 7 (GraphPad software, San Diego, CA, USA). A Shapiro–Wilk test was performed to determine the normality of each dataset, and all data were not normally distributed. First, correlation between the PIK3CA mutation to the IHC or RISH marker expressions were examined through the Mann–Whitney U test or Fisher’s exact test. Second, the relevance between histologic grade and IHC/RISH marker expression/PIK3CA mutation were analyzed by Fisher’s exact test or Pearson’s Chi-square test or the Kruskal–Wallis H test with the Dunn–Bonferroni post-hoc test. Third, the relation between PIK3CA mutation/IHC/RISH marker expression and histopathological/clinical features including histological tumor type, mitotic count, age of dogs, size of tumor, and spay was tested through the Mann–Whitney U test or Fisher’s exact test or Kruskal–Wallis H test with the Dunn–Bonferroni post-hoc test or Cramer’s V test or linear regression analysis. *p*-value below 0.05 was considered to be statistically significant.

## 3. Results

### 3.1. Histopathological Features of Samples

A total of 83 samples derived from canine mammary gland was included in the present study. Histopathological features of the samples are presented in Table 1. In 7/24 (29.2%) of high-grade carcinomas, neoplastic cells showed lymphatic invasion.

### 3.2. Frequency of PIK3CA H1047R Mutation and Relevance to Marker Expressions

**Figure 1:**
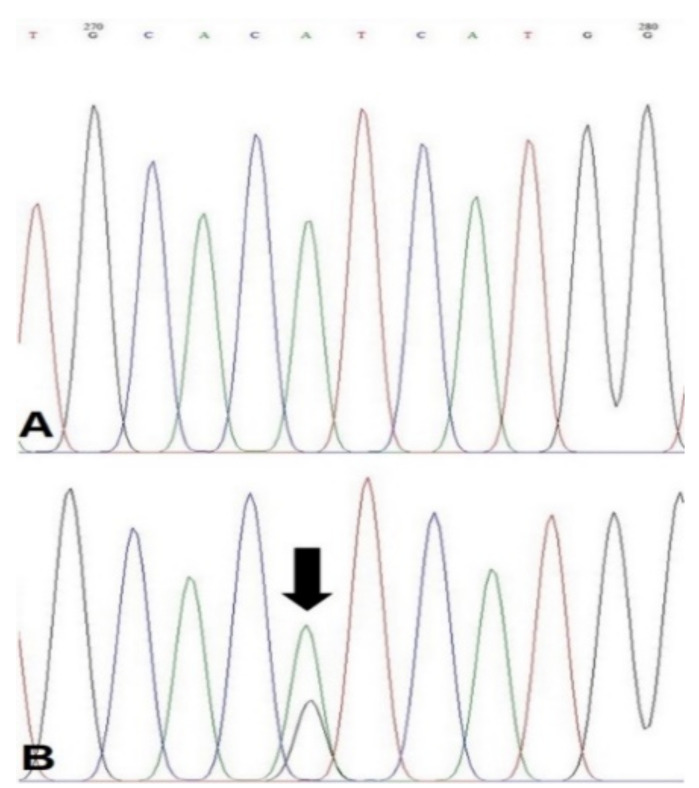
Sequence analysis for exon 20 of PIK3CA in canine mammary tumors. (**A**) Wild-type sequence of PIK3CA exon 20, and (**B**) mutant allele for missense mutation of PIK3CA (c.3140A > G) is detected (arrow).

PIK3CA H1047R mutation was found in 12% (10/83) of total samples and 14.3% (10/70) of tumor samples (Figure 1). The PIK3CA H1047R missense mutation found in each histological grade is presented in Table 2.

PIK3CA mutation status was not correlated to histological grade. When comparing PIK3CA-mutated tumors with PIK3CA-wild-type tumors, no remarkable differences were found in PIK3CA, Akt, Akt2, and p-Akt expression. Expression of PTEN was remarkably higher in PIK3CA-mutated tumors than those in PIK3CA-wild-type tumors (Figure S1; Mann–Whitney U test, *p* = 0.005).

### 3.3. Controls for IHC

In canine normal kidney tissue, PIK3CA and PTEN were detected in renal tubules, but not in glomeruli, and Akt was observed in both renal tubules and glomeruli, being the same expression patterns in humans (Figure S2). p-Akt was expressed in a few neoplastic endothelial cells in canine hemangiosarcoma (Figure S2). None of the cells in negative control slides exhibited positive signals, confirming the specificity of IHC in the present study (Figure S2).

### 3.4. Correlation of Histological Grade with PIK3CA H1047R Mutation and IHC/RISH Marker Expressions

**Figure 2:**
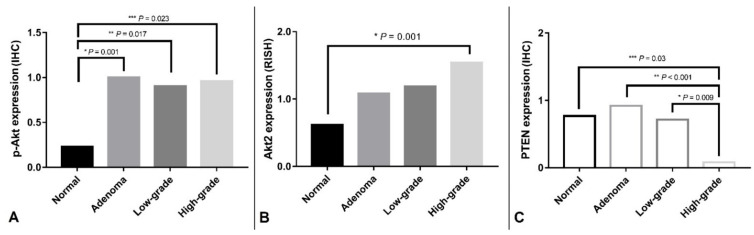
Bar charts for p-Akt, PTEN, and Akt2 expression in normal mammary gland and mammary tumor samples. (**A**) Expression of p-Akt was remarkably high in mammary adenoma, low-grade carcinoma, and high-grade carcinoma in comparison to normal mammary gland. (**B**) Akt2 was significantly upregulated in high-grade carcinoma than those in normal mammary glands. (**C**) Downregulation of PTEN was significant in high-grade carcinomas than in normal mammary gland, mammary adenoma, and low-grade carcinoma. IHC: Immunohistochemistry, RISH: RNA In situ hybridization. *, **, ***: Statistically significant.

Among the components of the PI3K/Akt/PTEN pathway, PIK3CA, Akt, Akt2, p-Akt, PTEN-p-Akt, Akt2 and PTEN presented remarkable differences between the four histological grades (Kruskal–Wallis H-test, Akt2, *p* = 0.014; p-Akt, *p* = 0.02; PTEN, *p* < 0.001). The results of IHC and RISH are presented in Figure 2.

**Figure 3:**
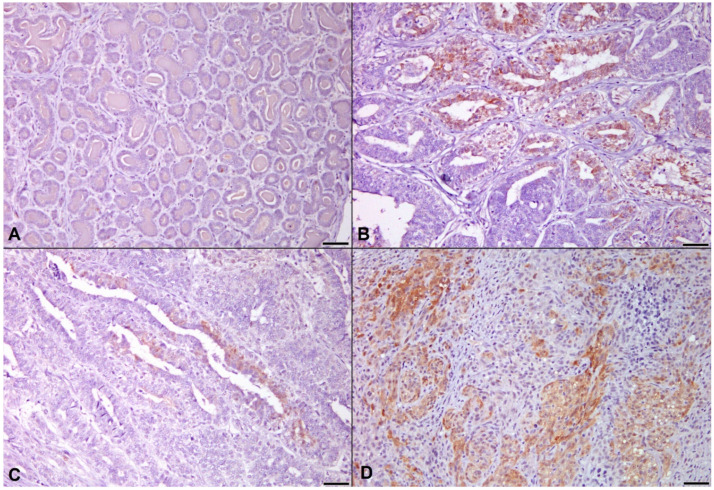
Immunohistochemistry for p-Akt. (**A**) Mammary gland hyperplasia. Expression of p-Akt was barely detected. (**B**) Mammary adenoma. p-Akt was found in both the nucleus and cytoplasm of neoplastic epithelial cells. (**C**) Mammary carcinoma, low-grade. Higher expression of p-Akt was observed than in normal mammary gland. (**D**) Mammary carcinoma, high-grade. Intense positive staining for p-Akt was shown in many neoplastic epithelial cells, indirectly showing the increased Akt activity in more malignant tumors. Immunohistochemistry, counterstained with 100% Gill’s hematoxylin. Original magnification 200×. Bar = 50 μm.

In the post-hoc test, increase of p-Akt was remarkable in adenoma, low-grade carcinoma, and high-grade carcinoma than those in normal mammary gland (Figure 3, *p* = 0.001; *p* = 0.017; *p* = 0.023, respectively).

**Figure 4:**
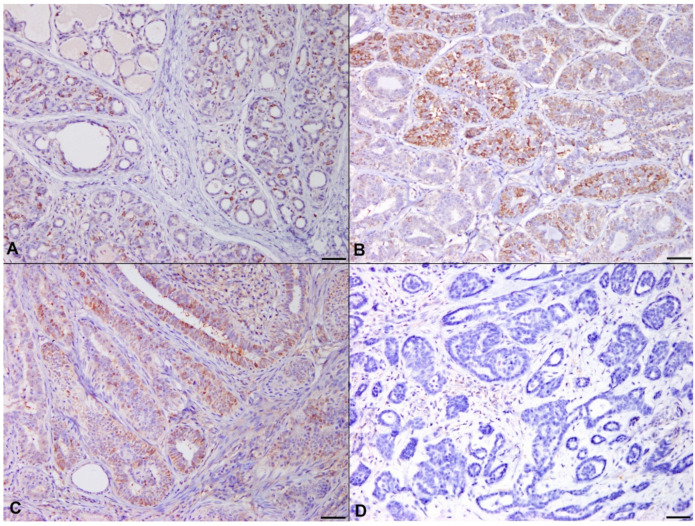
Immunohistochemistry for PTEN. (**A**) Normal mammary gland. Positivity for PTEN was found in many glandular epithelial cells. (**B**) Mammary adenoma. Intense cytoplasmic staining was observed in benign neoplastic cells. (**C**) Mammary carcinoma, low-grade. PTEN level was maintained at a similar level to those in normal mammary gland and mammary adenoma. (**D**) Mammary carcinoma, high-grade. Expression of PTEN was remarkably lost in invasive neoplastic cells, implying that loss of tumor suppressor PTEN contributes to malignant progression in canine mammary tumors. Immunohistochemistry, counterstained with 100% Gill’s hematoxylin. Original magnification 200×. Bar = 50 μm.

Downregulation of PTEN was significant in high-grade carcinoma compared with those in normal mammary gland, adenoma, and low-grade carcinoma (Figure 4, *p* = 0.03; *p* < 0.001; *p* = 0.009).

**Figure 5:**
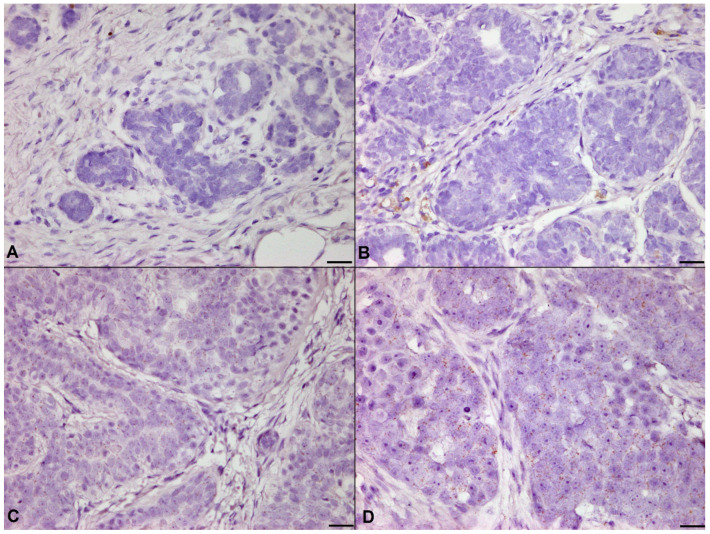
RNA in situ hybridization for Akt2. (**A**) Normal mammary gland. Brown dot represents a single transcript for Akt2, and a single or no RNA transcript was detected in normal glandular cells. (**B**) Mammary adenoma. Akt2 mRNA level was slightly increased or similar in mammary adenoma compared to normal mammary gland. (**C**) Mammary carcinoma, low-grade. Expression of Akt2 was comparable or mildly higher when compared to normal or mammary adenoma. (**D**) Mammary carcinoma, high-grade. Markedly higher expression of Akt2 transcripts was detected than those in the normal mammary gland. RNA in situ hybridization, counterstained with 50% Gill’s hematoxylin. Original magnification 400×. Bar = 20 μm.

All 83 samples were included in the RISH analysis as samples retained positive control UBC RNA level with scores of 3–5. Significant difference of Akt2 mRNA expression was also presented between four histologic grade groups (Figure 5, *p* = 0.001), and in the post-hoc test, Akt2 was found to be significantly increased in high-grade carcinoma than those in the normal mammary gland (*p* = 0.02).

### 3.5. Histopathological/Clinical Features and PIK3CA Mutation/Marker Expressions

In terms of histopathological features, PTEN expression was significantly lower in simple-solid carcinoma than those in complex adenoma and complex carcinoma (*p* < 0.001; *p* = 0.013, respectively). There were no significant correlations between PIK3CA mutation/marker expressions and spay, size of tumors, age of dogs, lymphatic invasion, and mitotic counts.

## 4. Discussion

In this study, we found that components of the PI3K/Akt/PTEN pathway—p-Akt, Akt2 and PTEN—were dysregulated and differentially expressed depending on histological grade. The current study also revealed that the PIK3CA H1047R mutation is a relatively frequent event in canine mammary tumors, and this mutation was mostly not correlated with downstream molecule expressions or histopathological/clinical features. In human breast cancers, PIK3CA mutation is associated with PTEN loss and Akt activation represented by p-Akt [41,42]. These dissimilarities may suggest that different mammary tumorigenic mechanism in humans and dogs, for example, activation of Akt may be PIK3CA-independent in canine mammary tumors, possibly phosphorylated by tyrosine or serine/threonine kinases [43]. However, the greater possibility for this discrepancy could be derived from insufficient numbers of PIK3CA-mutated samples (*n* = 10) in this study to correctly determine the feature of PIK3CA-mutated tumors. Because studies verifying canine PIK3CA H1047R are still limited and the numbers of cases in previous and the present study were relatively low, further large-scale studies are required to determine the frequency and the features of PIK3CA H1047R mutation in canine mammary tumors.

While PIK3CA H1047R missense mutation is a frequent event in canine mammary tumors in recent studies utilizing next-generation sequencing (29–32.8%) [27,28], mutation was observed in relatively low occurrence in the present study (14.3%). Low percentage of PIK3CA H1047R could be derived from the difference of sensitivity between next-generation sequencing and Sanger sequencing. As conventional Sanger sequencing needs 20% of allele frequency to detect the mutation and as mammary tumor is highly heterogenous [44], samples harboring low mutant allele could be false-negative in the present study.

Downregulation of PTEN in malignant tumors in this study corresponded to previous findings, emphasizing its role in canine mammary tumorigenesis [29,30,45]. PTEN loss is also a common feature in human cancer, derived from epigenetic silencing, mutation, and transcriptional repression [46]. Since PTEN is a multifaceted molecule associated with cell cycle, cell motility, genomic stability, and tumor microenvironment [46], and consistent results are shown in previous and current study, investigating PTEN in combination with other molecules could be beneficial to enhance our understanding of canine mammary tumors. Lower expression of PTEN in simple-solid carcinoma than in complex adenoma and complex carcinoma is also an interesting finding as has been shown in a previous study [29]. From the result of the previous and current study, it might be speculated that the reason why dogs with complex-type tumor survive longer than dogs with simple-type tumor [47] originates from the dysregulation of PTEN. From this point of view, PTEN itself could be a potential prognostic marker in canine mammary tumors. In addition, paradoxical higher expression of PTEN in PIK3CA-mutated tumors in the current study may be a true feature of PIK3CA-mutated tumors, however, it may be derived from the small sample size of PIK3CA-mutated tumors (*n* = 10) to correctly deduce the conclusion.

In the present study, the entire expression of Akt was similar in normal and tumor tissues, whereas contrasts in p-Akt and Akt2 expression were demonstrated in different histological grades. Thus, it could be concluded that although transcriptional and translational levels of Akt are not changed in tumors, activation of Akt and expression of Akt isoforms are dysregulated. Despite there being a conflicting result [29], activation of Akt is a seemingly early event in the transformation in canine mammary tumors as upregulation is shown from adenoma.

Moreover, as Akt2 has been demonstrated to promote tumor invasion and cell migration [48], significant upregulation of Akt2 transcription found in the present study may imply that overexpression of Akt2 in high-grade tumors constitutively function as a key factor for aggressiveness in canine mammary tumors. The previous [27,28] and the present study suggest that PIK3CA H1047R frequently occurs in canine mammary tumors. This mutational similarity to humans may shed light on new anticancer therapy in dogs. PI3K signal promotes the growth of estrogen receptor-positive breast cancer in an estrogen-independent manner, and blocking of PI3K inhibits the emergence of hormone-independent cancer cells [49]. PIK3CA mutation has been found to be common in canine mammary tumors and carcinogenesis of canine mammary tumor is estrogen-dependent with estrogen receptor expression [50]. Thus, PIK3CA could be suggested as a potential therapeutic target. For example, Alpelisib, which is a new anticancer drug and is clinically beneficial to PIK3CA-mutated breast cancer patients [49,51], might be a future medicine for dogs with mammary tumors.

## 5. Conclusions

Overall, the present study examined the dysregulation of PI3K/Akt/PTEN axis molecules and the PIK3CA H1047R mutation by utilizing Sanger sequencing in canine mammary tumors. PIK3CA, which has started to be highlighted in recent years, has undeniable worth in understanding molecular pathogenesis for canine tumors, and could be a potential therapeutic target in canine mammary tumors. Therefore, further large-scale studies investigating thee PIK3CA/Akt/PTEN pathway in canine mammary tumors are necessary.

## Figures and Tables

**Table 1:** Histopathological features of tissues samples.

Grade	Histological Type	Number of Samples
Normal	N/A	13
Adenoma	Ductal adenoma	12
	Intraductal papillary adenoma	2
	Complex adenoma	10
	Benign mixed tumor	1
Low-grade carcinoma	Simple carcinoma-tubulopapillary	1
	Simple carcinoma-tubular	2
	Complex carcinoma	17
	Carcinoma arising in a benign mixed tumor	1
High-grade carcinoma	Simple carcinoma-solid	17
	Simple carcinoma-tubulopapillary	2
	Simple carcinoma-tubular	1
	Simple carcinoma-comedocarcinoma	2
	Complex carcinoma	1
	Carcinosarcoma	1

**Table 2:** PIK3CA H1047R mutation in each histological grade.

	Grade	Percentage (Number of Samples)
PIK3CA-wild-type		88% (73/83)
PIK3CA-mutant	Normal (*n* = 13)	0% (0/83)
	Adenoma (*n* = 25)	4.8% (4/83)
	Low-grade carcinoma (*n* = 21)	6% (5/83)
	High-grade carcinoma (*n* = 24)	1.2% (1/83)

## Data Availability

The data presented in this study are available on request from the corresponding author.

## Supplementary Materials

The following are available online at https://www.mdpi.com/article/10.3390/ani11072079/s1, Figure S1: Akt, p-Akt, PTEN, and Akt expression in PIK3CA-mutated/wild-type tumors, Figure S2: Positive and negative controls for immunohistochemistry, Table S1: Information for primary antibodies and immunohistochemistry, Table S2: Evaluation criteria for immunohistochemistry and RNA in situ hybridization, Table S3: Raw clinical and experimental data for samples.
