# Supplementary material for: Dysregulation of PI3K/Akt/PTEN Pathway in Canine Mammary Tumor
Source: Animals (Basel). 2021 Jul 12;11(7):2079. doi: 10.3390/ani11072079 (PMC8300234; doi:10.3390/ani11072079)
Supplement: Supplementary file 1 [file animals-11-02079-s001.zip › animals-1265478-suppl. for publish/supplementary files.pdf]

animals

Article

Dysregulation of PI3K/Akt/PTEN Pathway in Canine Mammary Tumor

Soo-Hyeon Kim, Byung-Joon Seung, Seung-Hee Cho, Ha-Young Lim, Min-Kyung Bae and Jung-Hyang Sur *

Department of Veterinary Pathology, College of Veterinary Medicine, Konkuk University, Seoul 05025, Korea; windsl@naver.com (S.-H.K.); bjseung@naver.com (B.-J.S.); shchopa11@naver.com (S.-H.C.); hylim07@gmail.com (H.-Y.L.); mkbae4136@naver.com (M.-K.B.)

* Correspondence: jsur@konkuk.ac.kr

Citation: Kim, S.-H.; Seung, B.-J.; Cho, S.-H.; Lim, H.-Y.; Bae, M.-K.; Sur, J.-H. Dysregulation of PI3K/Akt/PTEN Pathway in Canine Mammary Tumor. *Animals* **2021**, *11*, 2079. https://doi.org/10.3390/ani11072079

Academic Editor: Stanislaw Dzimira

Received: 2 June 2021
Accepted: 10 July 2021
Published: 12 July 2021

Publisher's Note: MDPI stays neutral with regard to jurisdictional claims in published maps and institutional affiliations.

Copyright: © 2021 by the authors. Submitted for possible open access publication under the terms and conditions of the Creative Commons Attribution (CC BY) license (http://creativecommons.org/licenses/by/4.0/).

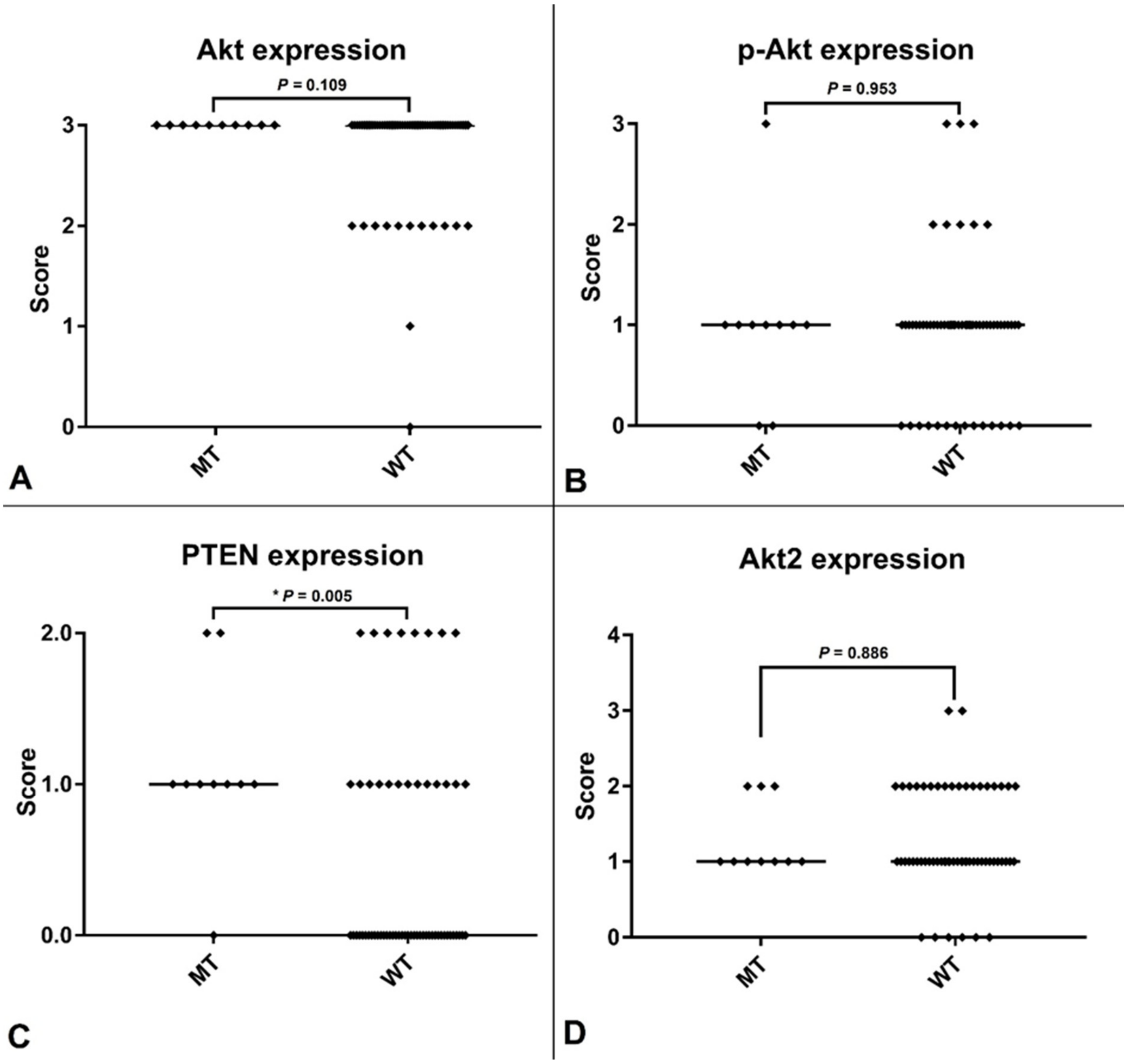

Figure S1. Akt, p-Akt, PTEN and Akt expression in PIK3CA-mutated / wild-type tumors. Each dot represents one case of samples. (A, B, D) Expression of Akt (immunohistochemistry), p-Akt (immunohistochemistry) and Akt2 (RNA in situ hybridization) is not significantly different in PIK3CA-mutated and PIK3CA-wild-type tumors. (C) In contrast, PTEN level is remarkably high in PIK3CA-mutated tumors than those in PIK3CA-wild-type tumors. MT: mutated tumors, WT: wild-type tumors.

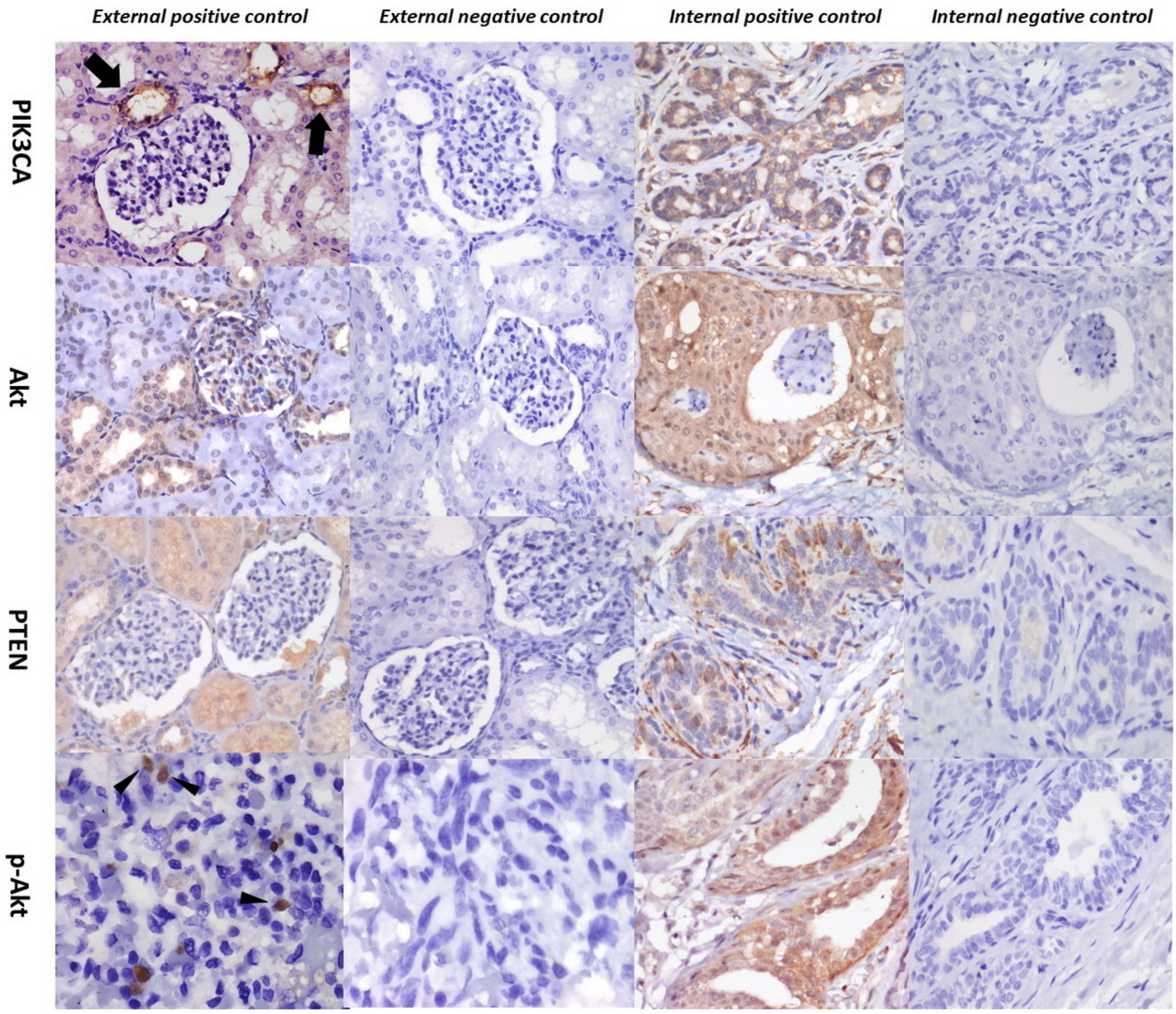

Figure S2. Positive and negative controls for immunohistochemistry. PIK3CA and PTEN exhibits positivity in renal tubules (arrow) but not in glomeruli. Akt is detected in both renal tubule and glomeruli. Expression of Akt is observed in renal tubules. A few of neoplastic endothelial cells show positivity to p-Akt (arrowhead). In internal positive controls, all markers show adequate staining in neoplastic mammary epithelial cells. None of external or internal negative control slides show positivity.

Table S1. Information for primary antibodies and immunohistochemistry.

Marker	Clonality	Supplier	Antigen retrieval	Dilution	Incubation
PIK3CA	Rabbit polyclonal	GeneTex	Citric acid, 8 min	1:4000	4°C, Overnight
Akt	Rabbit monoclonal (Clone: Y89)	Abcam	Citric acid, 8 min	1:400	4°C, Overnight
p-Akt	Rabbit polyclonal	CellSignaling	Citric acid, 30 min	1:50	4°C, Overnight
PTEN	Mouse monoclonal (Clone: A2B1)	SantaCruz	Citric acid, 20 min	1:200	4°C, Overnight

Table S2. Evaluation criteria for immunohistochemistry and RNA in situ hybridization.

Marker	Evaluation criteria
PIK3CA	Stained cell proportion<br>- 0 (<5%); 1 (5–25%); 2 (26–50%); 3 (>50%)<br><br>Intensity of immunostaining<br>- 0 (negative); 1 (weak); 2 (moderate); 3 (strong)<br><br>Total score = (Stained cell proportion) × (Intensity of immunostaining)<br>Total score: <4 (negative); ≥4 (positive)
Akt	0 (No staining)<br>1 (Positivity in <10% of cells)<br>2 (Positivity in 11–50% of cells)<br>3 (Positivity in >50% of cells)
p-Akt	0 (No staining)<br>1 (<20% of cells with mild to moderate staining intensity)<br>2 (20–50% of cells with mild to moderate staining intensity or <20% of cells with strong intensity)<br>3 (>50% of cells with mild to moderate staining intensity or >20% of cells with strong intensity)
PTEN	0 (Positivity in 0–25% of cells)<br>1 (Positivity in 25–50% of cells)<br>2 (Positivity in >50% of cells)
Akt2	0 (No staining or 1 dot/cell under a 40× objective lens)<br>1 (1–3 dots/cell under a 20–40× objective lens)<br>2 (4–10 dots/cell and no or very few clusters of dots under a 20–40× objective lens)<br>3 (>10 dots/cell and <10% of positive cells with dot clusters under a 20× objective lens)<br>4 (>10 dots/cell and >10% of positive cells with dot clusters under a 20× objective lens)
